# Novel multipurpose pod-intravaginal ring for the prevention of HIV, HSV, and unintended pregnancy: Pharmacokinetic evaluation in a macaque model

**DOI:** 10.1371/journal.pone.0185946

**Published:** 2017-10-05

**Authors:** James M. Smith, John A. Moss, Priya Srinivasan, Irina Butkyavichene, Manjula Gunawardana, Rob Fanter, Christine S. Miller, Debbie Sanchez, Flora Yang, Shanon Ellis, Jining Zhang, Mark A. Marzinke, Craig W. Hendrix, Amita Kapoor, Marc M. Baum

**Affiliations:** 1 Laboratory Branch, Division of HIV/AIDS Prevention, National Center for HIV/AIDS, Viral Hepatitis, STD, and TB Prevention, Centers for Disease Control and Prevention, Atlanta, Georgia, United States of America; 2 Department of Chemistry, Oak Crest Institute of Science, Monrovia, California, United States of America; 3 Libra Management Group, Decatur, Georgia, United States of America; 4 Department of Medicine, Johns Hopkins University, Osler, Baltimore, Maryland, United States of America; 5 Department of Pathology, Johns Hopkins University, Sheikh Zayed Tower, Baltimore, Maryland, United States of America; 6 Wisconsin National Primate Research Center, University of Wisconsin-Madison, Capitol Court, Madison, Wisconsin, United States of America; University of Pittsburgh, UNITED STATES

## Abstract

Globally, women bear an uneven burden for sexual HIV acquisition. Results from two clinical trials evaluating intravaginal rings (IVRs) delivering the antiretroviral agent dapivirine have shown that protection from HIV infection can be achieved with this modality, but high adherence is essential. Multipurpose prevention technologies (MPTs) can potentially increase product adherence by offering protection against multiple vaginally transmitted infections and unintended pregnancy. Here we describe a coitally independent, long-acting pod-IVR MPT that could potentially prevent HIV and HSV infection as well as unintended pregnancy. The pharmacokinetics of MPT pod-IVRs delivering tenofovir alafenamide hemifumarate (TAF_2_) to prevent HIV, acyclovir (ACV) to prevent HSV, and etonogestrel (ENG) in combination with ethinyl estradiol (EE), FDA-approved hormonal contraceptives, were evaluated in pigtailed macaques (*N* = 6) over 35 days. Pod IVRs were exchanged at 14 days with the only modification being lower ENG release rates in the second IVR. Plasma progesterone was monitored weekly to determine the effect of ENG/EE on menstrual cycle. The mean *in vivo* release rates (mg d^-1^) for the two formulations over 30 days ranged as follows: TAF_2_ 0.35–0.40; ACV 0.56–0.70; EE 0.03–0.08; ENG (high releasing) 0.63; and ENG (low releasing) 0.05. Mean peak progesterone levels were 4.4 ± 1.8 ng mL^-1^ prior to IVR insertion and 0.075 ± 0.064 ng mL^-1^ for 5 weeks after insertion, suggesting that systemic EE/ENG levels were sufficient to suppress menstruation. The TAF_2_ and ACV release rates and resulting vaginal tissue drug concentrations (medians: TFV, 2.4 ng mg^-1^; ACV, 0.2 ng mg^-1^) may be sufficient to protect against HIV and HSV infection, respectively. This proof of principle study demonstrates that MPT-pod IVRs could serve as a potent biomedical prevention tool to protect women’s sexual and reproductive health and may increase adherence to HIV PrEP even among younger high-risk populations.

## Introduction

Sexually transmitted infections (STIs), including human immunodeficiency virus (HIV), and unintended pregnancy represent a major global burden for women particularly in resource-limited regions.

The global response to the HIV epidemic has resulted in the number of new infections being halved in 2012 since the peak in 1996 [1], largely resulting from scale-up of prevention and treatment efforts. In Fast-Track, the UNAIDS has set the aggressive target of 500,000, or fewer, new annual infections by 2020, a 75% reduction from 2010 numbers, and ending the AIDS epidemic by 2030 [2]. However, recent statistics suggest that a prevention gap has been reached, with the number of annual, new HIV infections stalling around 1.9 million since 2010 [3]. To meet the ambitious UNAIDS Fast-Track goals, highly effective biomedical prevention modalities for HIV prevention will be required.

Herpes simplex virus type 2 (HSV-2), the serotype most commonly associated with genital herpes, and HIV form the basis for two intersecting epidemics, where morbidity from one virus facilitates the transmission and pathogenesis by the other [4–7]. A systematic meta-analysis of longitudinal studies found HSV-2 incidence to be associated with a two- to five-fold increased risk of HIV acquisition among both men and women [8, 9]. Seroprevalence rates of HSV-2 are highest in developing countries, in particular throughout Africa and the Americas [10], and can range from 60% to 80% in young adults [11]. Infection by HSV-2 can lead to inflammation of the vaginal mucosa, increasing susceptibility to HIV infection and virus replication at the point of entry and potentially reducing topical PrEP effectiveness [12]. Consequently, interventions against HSV-2 may have a key role in HIV prevention initiatives [13].

There is accumulating clinical trial evidence suggesting that oral pre-exposure prophylaxis (PrEP) regimens based on the prodrug tenofovir disoproxil fumarate (TDF) can be effective at preventing HIV transmission [14–20], but low adherence to product use also has contributed to a number of negative trial results [21]. Adherence to therapy is known to be inversely related to dosing period [22–25]. The compliance burden associated with frequent dosing can be reduced with long-acting drug formulations, such as intravaginal rings (IVRs) [26] as well as implantable and injectable formulations [27]. In addition, multipurpose prevention technologies (MPTs) represent an integrated biomedical approach aimed at providing dual protection [28–30], usually from HIV infection and unintended pregnancy. The MPT strategy exploits synergies in demand for protection [31], significantly increasing the likelihood of increased uptake relative to single-purpose products [32].

Two phase 3, randomized, double-blind, placebo-controlled trials (MTN-020–ASPIRE and IPM 027–The Ring Study) recently evaluated a monthly IVR delivering the non-nucleoside HIV reverse-transcriptase inhibitor dapivirine. The trials enrolled 2,629 and 1,959 women, respectively, between the ages of 18 and 45 years in Malawi, South Africa, Uganda, and Zimbabwe and demonstrated that an IVR delivering an ARV agent can reduce the risk of HIV acquisition by as much as 56% in highly adherent users. However, it also showed that certain subgroups, particularly young women between 18–21 years of age, did not adhere to IVR use and, consequently, were not protected from HIV infection [33]. An MPT IVR against HIV, HSV, and unintended pregnancy may help overcome adherence issues.

The primary purpose of this proof-of-concept study was to describe an innovative, triple-purpose MPT IVR (termed “pod-IVR”) delivering the ARV drug tenofovir alafenamide hemifumarate (TAF_2_) in combination with the antiherpetic drug acyclovir (ACV) and etonogestrel (ENG)-ethinyl estradiol (EE), an established progestin-estrogen combination for contraception. The pharmacokinetics of the MPT IVR were evaluated in pigtailed macaques and demonstrated that all four agents could be delivered at independently controlled release rates to provide vaginal mucosal, tissue, and systemic drug concentrations required for therapeutic efficacy.

## Materials and methods

### Materials

Tenofovir alafenamide hemifumarate (TAF_2_) [34] was kindly provided by Gilead Sciences, Inc. (Foster City, CA) under a Material Transfer Agreement (MTA) dated 11/08/13. The remaining active pharmaceutical ingredients (APIs) and excipients were purchased from commercial sources. Stable isotope labeled standards were purchased from Moravek Biochemicals, Inc. (Brea, CA) and Santa Cruz Biotechnology, Inc. (Dallas, TX). Polyvinyl alcohol (PVA) with a mean molecular weight (*M*_*w*_) 85,000–124,000 kD (98–99% hydrolyzed) was obtained from Sigma-Aldrich (St. Louis, MO). All other reagents were obtained from Sigma-Aldrich, unless otherwise noted.

### Fabrication of combination pod-intravaginal rings

Macaque-sized [35] polydimethylsiloxane (PDMS, silicone) pod-IVRs were prepared in a multi-step process that has been described in detail elsewhere [36–39]. Two configurations of pod-IVRs were manufactured, as summarized in Table 1, where Configuration A was designed for rapid ENG release and Configuration B was designed for slow ENG release.

**Table 1:** Drug loading in pod-IVRs used in pigtailed macaque studies.

	IVR API loading (mg)^a^^,^^b^	
API	Configuration A^c^	Configuration B^d^
TAF_2_	45.0 ± 1.8	43.9 ± 1.4
ACV	44.2 ± 2.2	46.0 ± 1.5
ENG	22.0 ± 0.2	10.5 ± 0.2
EE	11.1 ± 0.1	10.5 ± 0.2

^a^represents total drug loading in IVR

^b^mean ± *SD*

^c^rapid-releasing ENG

^d^slow-releasing ENG

### *In vitro* studies

All *in vitro* release studies were designed to mimic sink conditions using methods reported previously [36]. Briefly, the IVRs were placed in a simplified vaginal fluid simulant (VFS) [40] dissolution media (100 mL) consisting of 25 mM acetate buffer (pH 4.2) with NaCl added to achieve 220 mOs. The vessels were agitated in an orbital shaker at 37 ± 2°C and 60 rpm. In some cases, the media was replaced daily. Aliquots (100 μL) were removed at predetermined timepoints and were replaced with an equal volume of dissolution media. Samples were stored at -20°C prior to analysis.

The concentration of TAF_2_, its hydrolysis product tenofovir (TFV) and metabolite Met Y [41, 42], ACV, ENG, and EE was measured by HPLC with UV detection (1100 Series, Agilent Technologies, Santa Clara, CA). For measurement of residual TAF_2_, as well as TFV and Met Y, ACV, ENG, and EE in used IVRs, the mobile phase was composed of A: 1% acetic acid + 3% acetonitrile in water and B: acetonitrile. The gradient program was 0% B (2 min); 0–25% B (2 min); 25% B (1 min); 25–50% B (3 min); and 50% B (6 min) at a flow rate of 0.5 mL min^-1^. A Waters (Milford, MA) Atlantis T3 C18 column (2.1 × 100 mm; 5 μm; 100A) was used as the stationary phase. The retention times were: TAF_2_, 7.71 min; Met Y, 5.86 min; TFV, 1.08 min.; ACV, 1.29 min; EE, 10.82 min; ENG, 11.99 min. For *in vitro* release studies, TAF_2_, TFV, and ACV were measured using the same method. For *in vitro* release studies of ENG and EE, the mobile phase consisted of acetonitrile:water in the ratio of 50:50 (vol/vol), and a Phenomenex (Torrance, CA) Kinetex XB-C18 column (2.1 × 100 mm; 2.6 μm; 100A) was used as the stationary phase. The retention times were: EE, 2.37 min; ENG, 4.04 min. For all methods, detection wavelengths were 260 nm (TAF_2_, Met Y, TFV, and ACV), 240 nm (ENG), and 280 nm (EE).

### Nonhuman primate studies

#### Ethics statement

All pigtailed macaques (*Macaca nemestrina*) used in this study were housed at the Centers for Disease Control and Prevention (CDC, Atlanta, GA), and the work was performed as described in the animal use research protocol (2445SMIMONC) approved by the CDC Institutional Animal Care and Use Committee. The facility is AAALAC accredited (AAALAC #00052 and PHS assurance #A4365-01) and animal husbandry and enrichment is performed by the Comparative Medicine Branch (CMB) under the direct supervision of the attending veterinarians and the animal enrichment coordinator. All efforts are made to pair house compatible animals by species and gender whenever possible. Cage sizes comply with the Animal Welfare Act and as outlined in the CMB Environmental Enhancement plan (dimensions: 30” wide × 30” deep × 30” tall). Macaques were monitored twice daily for physical and behavioral health by trained CMB staff and undergo quarterly physical examinations. Animals were routinely screened for enteric pathogens, and monitored for weight loss and behavioral abnormalities (lethargy, loss of appetite, self-mutilation). All treatments are at the discretion of the attending veterinarians and trained CMB staff. The investigator is consulted if a condition cannot be managed medically and if so, animals are humanely euthanized to prevent further trauma in accordance with the 2000 Report of the AVMA Panel on Euthanasia (http://www.avma.org/issues/animalwelfare/euthanasia.pdf). Standard enrichment for nonhuman primates includes access to objects to manipulate in the cage, swings or perches, a variety of food supplements such as fresh fruits, seeds, and vegetables, foraging and/or task-oriented feeding methods, and interaction with animal caretakers.

All animals in this study were previously enrolled in CDC IACUC approved efficacy studies and were infected with SHIV162P3 and subsequently released to protocol 2445SMIMONC and scheduled for euthanasia. The animals used in this study were only available for a short time. For terminal PK studies we only use SHIV-infected animals slated for necropsy. Sacrificing healthy uninfected animals for terminal PK studies is not approved under the CDC IACUC protocol that was used for this study, especially given the high cost and restricted availability of female pigtailed macaques. At the end of this study all macaques were euthanized (intravenous pentobarbital) in accordance with the 2000 Report of the AVMA Panel on Euthanasia (http://www.avma.org/issues/animalwelfare/euthanasia.pdf).

#### Pharmacokinetic study

**Fig 1:**
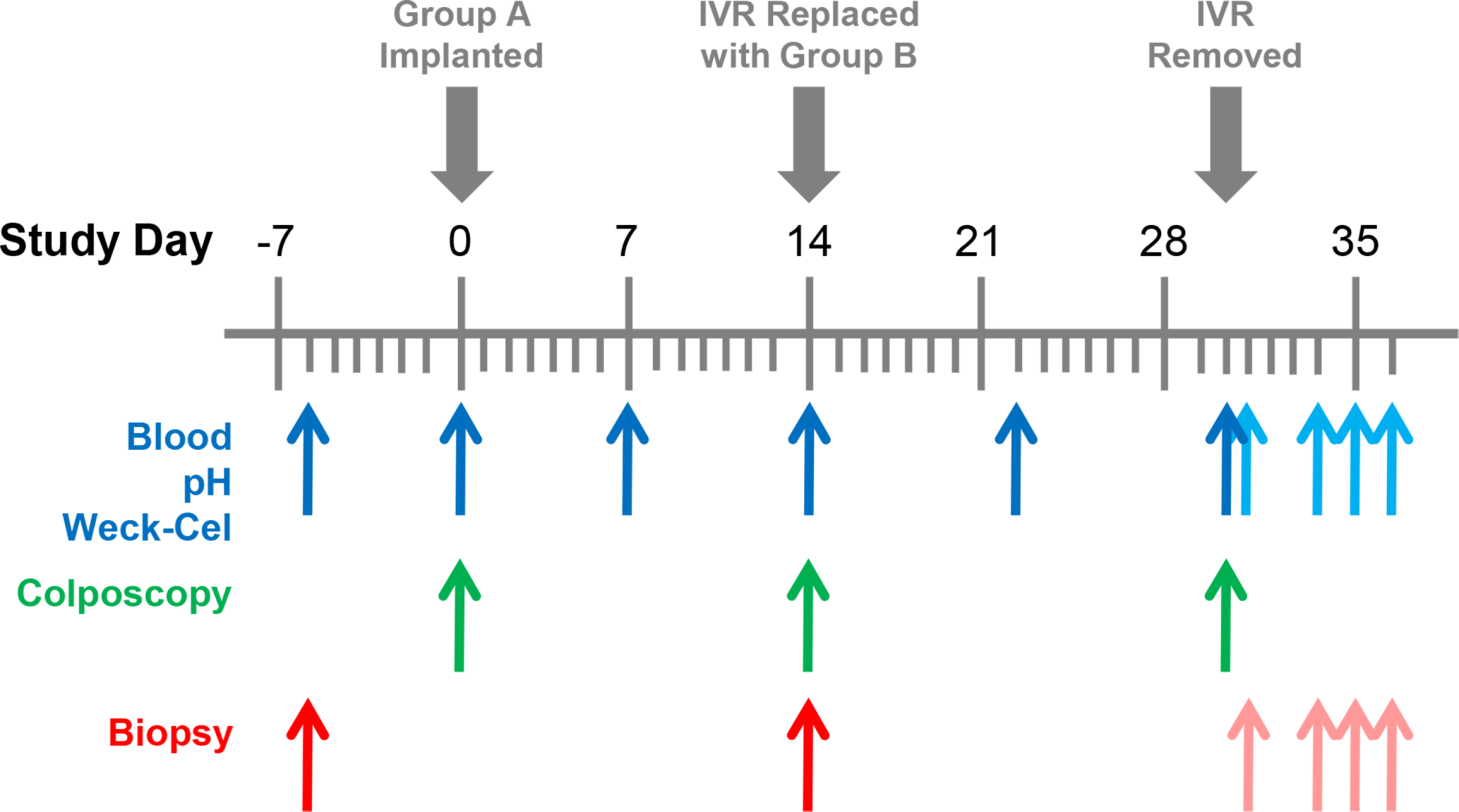
Pigtailed macaque TAF2-ACV-ENG-EE pod-IVR study timelines and biological sample collection points (*N* = 6). Configuration A pod-IVRs (rapid-releasing ENG) were inserted on Day 0 and replaced on Day 14 with Configuration B pod-IVRs (slow-releasing ENG). The Configuration B IVRs were removed on Day 30 and animals were euthanized on Day 34 (animal ID BB495 and DC42), Day 35 (animal ID PHz1 and PPk2), and Day 36 (animal ID PEc2 and BB0539). Blue arrows, in order of collection, blood, pH, and vaginal fluid (two Weck-Cel samples per time point—two proximal and two distal to the IVR). Green arrows, colposcopy examination (right after blood collection). Red arrows, vaginal tissues (six pinch biopsies per time point—three proximal and three distal to the IVR). Complete vaginal tracks were collected at necropsy. Pale blue and pale red arrows correspond to sample collection points with the IVR removed.

The pharmacokinetic (PK) study was carried out at the Centers for Disease Control and Prevention (CDC) under approved CDC Institutional Animal Care and Use Committee protocol 2445SMIMONC, and standard guidelines according to the *Guide for the Care and Use of Laboratory Animals* (DHEW No. NIH 86–23). The study timeline and biological sample collection points are shown in Fig 1 and employed published protocols [39, 43]. Briefly, six sexually mature female pigtailed macaques (*Macaca nemestrina)* were used in the study. Configuration A pod-IVRs (rapid-releasing ENG) were inserted on Day 0 into the posterior vagina and were replaced on Day 14 with Configuration B pod-IVRs (slow-releasing ENG) and remained in place for another 16 days. The two configurations were designed to test fast- and slow-releasing formulations of ENG sequentially.

Animals were humanely euthanized using techniques recommended by the American Veterinary Medical Association Guidelines on Euthanasia, 2013, and in accordance with CDC-Atlanta IACUC Policy 016 on Euthanasia. Necropsies were performed in groups of two on Days 34, 35, and 36. At necropsy, the complete vaginal tracts (ca. 4 × 6 cm) were collected and sectioned into uniform 5 × 7 grids (cervix to introitus in the smaller dimension) and the sections (ca. 8 mm in dia. or square) preserved for analysis. Multiple sections (ca. 12–15) were combined for CD4^+^ and CD4^-^ cell isolation using published methods [44, 45]. CD4^+^ and CD4^-^ cells were isolated from inguinal and iliac lymph node tissues using analogous methods.

### Safety measures

Rudimentary product safety was evaluated by clinical observations, cage-side observations (twice daily), and vaginal pH.

### *In vivo* pod-IVR drug release rates

Used IVRs were analyzed for residual drug content using published methods [39]. The HPLC methods were the same as those used to analyze aliquots from the *in vitro* studies.

### Bioanalysis of *in vivo* samples

Concentrations of TAF_2_, TFV, ACV, ENG, and EE in vaginal fluids, vaginal tissue homogenates, cellular lysates (CD4^+^ and CD4^-^ cells isolated from vaginal and lymph node tissues), and plasma were measured by LC-MS/MS using published methods [45, 46] and the methods described below. The analyses were performed at the following institutions: TFV and TFV-DP, Johns Hopkins University; ACV, ENG, and EE in cervicovaginal fluid (CVF), Oak Crest Institute; and EE in plasma, Wisconsin National Primate Research Center. The analytical ranges in the above sample matrices are presented in Table 2. Progesterone plasma concentrations were analyzed by the Wisconsin National Primate Research Center using a published method [47]. All new bioanalysis methods are described in detail in the Supporting Information (S1 File).

**Table 2:** Analytical ranges for biological samples.

Sample matrix (units)	TAF^a^	TFV	TFV-DP^b^	ACV	ENG	EE
Vaginal fluid (ng sample^-1^)	10–10,000	10–5,000	NA^d^	100–10,000	5–1,025	0.05–3^c^
Vaginal tissue, homogenate (ng sample^-1^)	NA^d^	0.05–50	50–1,500	1–5,000	NA^d^	NA^d^
Vaginal tissue, intracellular (ng sample^-1^)	NA^d^	NA^d^	50–1,500	NA^d^	NA^d^	NA^d^
Plasma (ng mL^-1^)	NA^d^	0.31–1,000	NA^d^	1–50	0.25–5.0	0.019–0.6

The low number in the range represents the analytical lower limit of quantitation (LLOQ).

^a^analyzed as the free-base (TAF), not the hemifumarate salt (TAF_2_)

^b^fmol sample^-1^

^c^measured by ELISA

^d^NA, not applicable

### Pharmacokinetic analysis

Pharmacokinetic parameter values were determined by noncompartmental analysis (NCA) using Phoenix WinNonlin 6.4 (Pharsight Corporation, Sunnyvale, CA). The NCA was run using the linear trapezoidal rule for increasing concentration data and the logarithmic trapezoidal rule for decreasing concentration data (linear up and log down) as the calculation method, and concentration values below the corresponding LLOQs (*C*_*LLQ*_) were treated as:

$$C_{LLQ}=\frac{\mathrm{LLOQ}\;\mathrm{of}\;\mathrm{assay}}{2\times \left(\mathrm{median}\;\mathrm{sample}\;\mathrm{mass}\right)}$$ (1)

### Statistical analysis

Data were analyzed using GraphPad Prism (version 7.00, GraphPad Software, Inc., La Jolla, CA). Unpaired comparisons were tested using one-way ANOVA for three or more groups and unpaired *t* tests with Welch’s correction for two groups. We tested for paired differences among conditions using the Friedman test with Dunn’s post test for multiple comparisons as well as Wilcoxon matched-pairs signed rank test where appropriate. The statistical hypothesis test used to compare each dataset is specified below. Statistical significance is defined as *P* < 0.05.

## Results

### *In vitr*o studies

**Fig 2:**
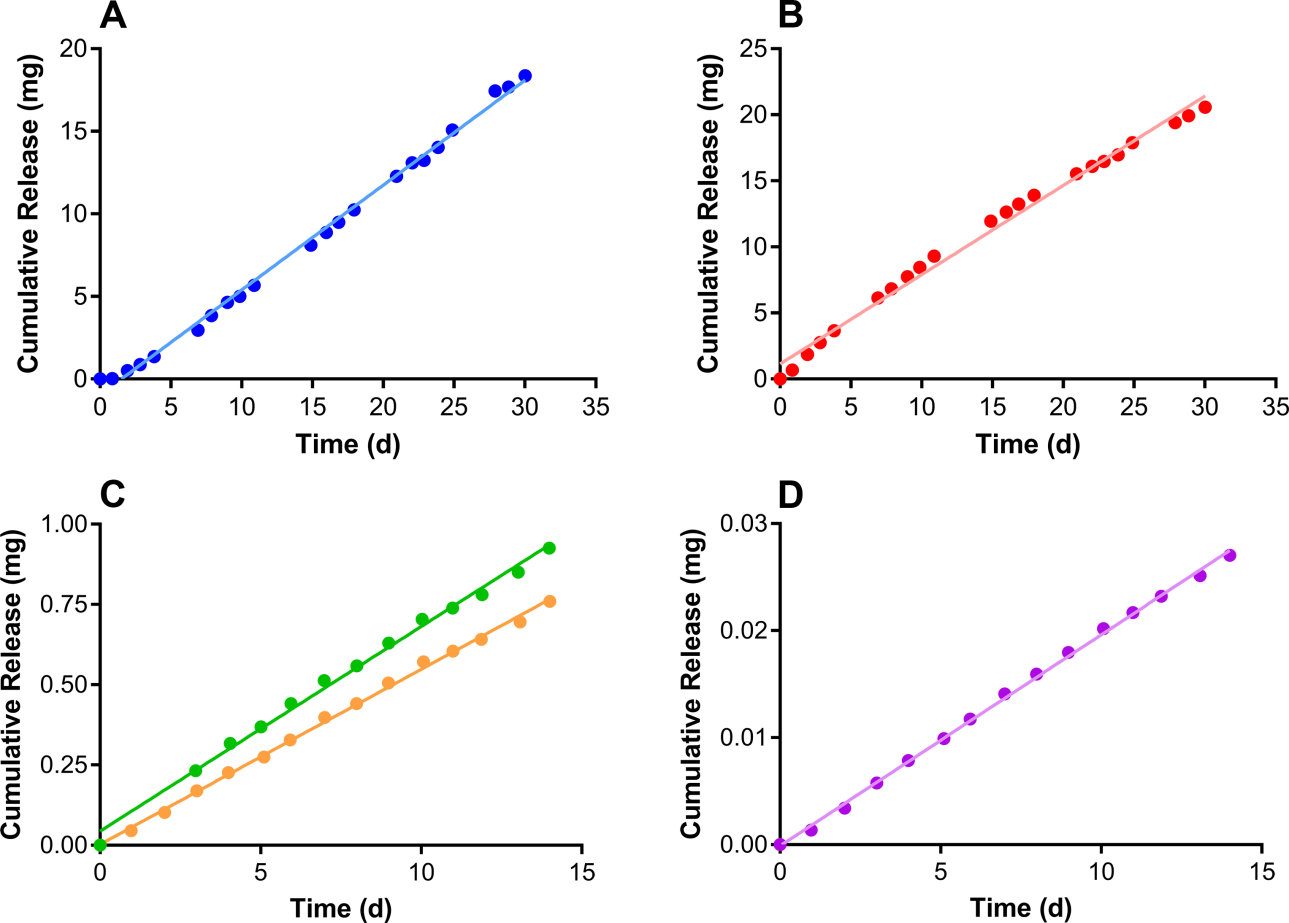
TAF_2_-ACV-ENG-EE pod-IVR *in vitro* release kinetics. Circles correspond to means (N = 4–6) and lines correspond to linear regressions. (A) TAF_2_. (B) ACV. (C) ENG: green, pod-IVR Configuration A; orange, pod-IVR Configuration B. (D) EE.

*In vitro* cumulative release profiles for the Configuration A and Configuration B IVR formulations (Fig 2) exhibited linear, sustained drug release (TAF_2_, *R*^*2*^ = 0.997; ACV, *R*^*2*^ = 0.991; ENG-A, *R*^*2*^ = 0.994; ENG-B, *R*^*2*^ = 0.998; EE, *R*^*2*^ = 0.999), as is typical for pod-IVRs [26, 36, 37, 39, 43, 48, 49]. The daily release rates obtained from the slopes of the cumulative release profiles are presented in Table 3. The *in vitro* release rates for highly water-insoluble compounds, such as ENG and EE, often are not predictive of *in vivo* release rates, as seen here. Previous pod-IVR studies have shown that while *in vitro* studies are useful for product quality control, *in vitro-in vivo* correlations (IVIVCs) of unity are not usually observed [39, 49].

**Table 3:** *In vitro* and *in vivo* daily release rates from Configuration A and Configuration B MPT pod-IVRs.

Drug	*In vitro* release rate (mg day^-1^)^b^	*In vivo* release rate (mg day^-1^)^b^
Configuration A^a^		
TAF_2_	0.64 ± 0.01	0.40 ± 0.07
ACV	0.68 ± 0.01	0.70 ± 0.10
ENG	0.063 ± 0.8×10^−3^	0.63 ± 0.14
EE	2.0×10^−3^ ± 0.03×10^−3^	0.033 ± 0.021
Configuration B^a^		
TAF_2_	0.64 ± 0.01	0.35 ± 0.08
ACV	0.68 ± 0.01	0.56 ± 0.09
ENG	0.055 ± 0.8×10^−3^	0.053 ± 0.014
EE	2.0×10^−3^ ± 0.03×10^−3^	0.078 ± 0.008

^a^ *N* = 6

^b^ mean ± *SD*

### *In vivo* drug release rates

**Fig 3:**
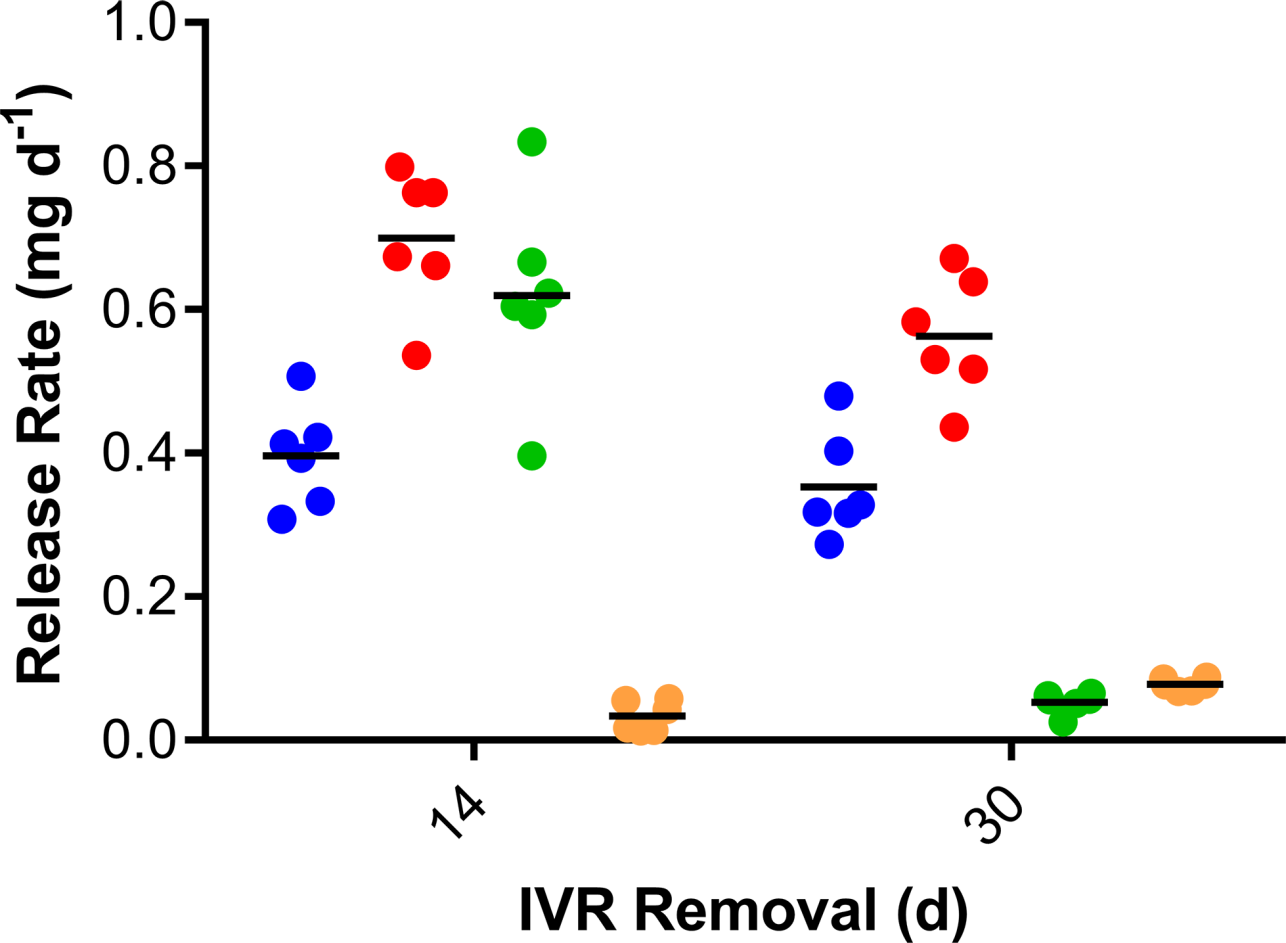
TAF2-ACV-ENG-EE pod-IVR *in vivo* daily drug release rates based on residual drug measurements on used IVRs. Blue circles, TAF_2_; red circles, ACV; green circles, ENG; orange circles, EE. Horizontal lines represent means.

The mean daily *in vivo* drug release rates for Configuration A (removed on Day 14) and Configuration B (removed on Day 30) pod-IVRs are provided in Table 3 and Fig 3. Release rates are based on the residual drug mass remaining in the used IVRs and the assumption, supported by *in vitro* data (Fig 2) that drug release is linear over the period of IVR use. Importantly, >95% of the residual TAF_2_ in the used IVR pods was present as the prodrug by HPLC; i.e., no significant prodrug hydrolysis was observed following two weeks of use *in vivo*. The Configuration A and Configuration B drug *in vivo* release rates (with the exception of ENG) were compared using an unpaired *t* test with Welch’s correction: TAF_2_, not significantly different (*P* = 0.3316); ACV, significantly different (*P* = 0.0276); and EE, significantly different (*P* = 0.0002).

**Fig 4:**
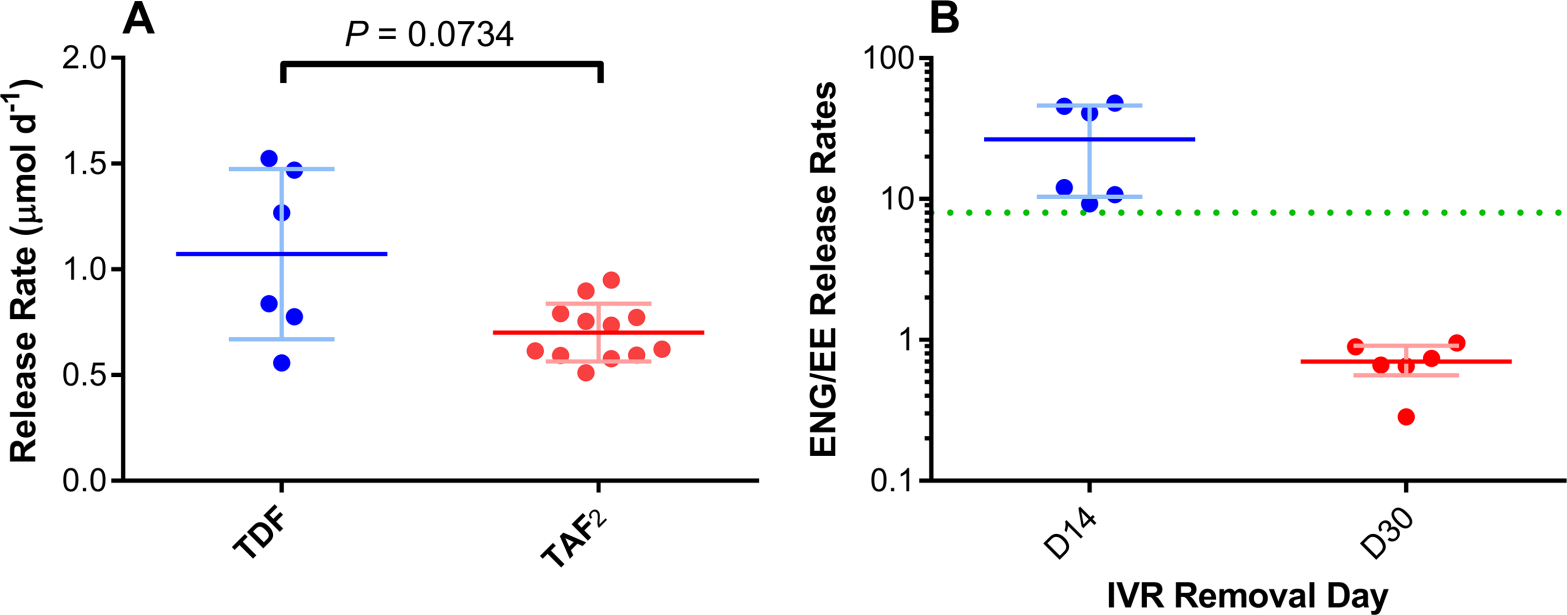
*In vivo* IVR drug release rates in the context of previous studies. **(A) Molar daily *in vivo* release of TDF from a previous pod-IVR study in pigtailed macaques [39] compared to TAF**_**2**_ **release rate in the present study.** An unpaired *t* test with Welch’s correction shows that while the release rates are not statistically significantly different (*P* = 0.0734), they are similar, with the mean TAF_2_ release rate (red horizontal bar) being lower than the mean TDF release rate (blue horizontal bar) from the TDF-FTC pod-IVRs. Each circle corresponds to an individual datum; pale error bars correspond to standard deviations from the mean. (B) Paired ENG:EE *in vivo* release rate ratios for Configuration A (blue) and Configuration B (red) pod-IVRs. The ratios span the *in vivo* ENG:EE release rate ratio for the NuvaRing® in women (green broken horizontal line). Each circle corresponds to an individual datum; horizontal lines, means; pale error bars, standard deviations from the mean.

The TAF_2_ *in vivo* molar release rates were not significantly different (*P* = 0.0734) from TDF release rates, also in pigtailed macaques, from a previous study using TDF-FTC pod-IVRs [39] (Fig 4A) allowing meaningful comparison of PK parameters for both TFV prodrugs. The median molar TAF_2_ release rate was 64% of the median TDF release rate in these studies. An important advantage of the pod-IVR is that drugs are physically isolated from one another and each pod releases its payload at an independently controlled rate [36], making the comparison possible across studies.

The high (Configuration A) and low (Configuration B) ENG *in vivo* release rates and the consistently low EE release rates allowed a wide range of *in vivo* ENG:EE release rate ratios to be attained (Fig 4B). These ratios bracket the corresponding value (*in vivo* ENG:EE release rate ratio = 8) from the NuvaRing® in women, as shown in Fig 4B by the green broken line.

### Safety measures

All IVRs were retained and there were no observations of abnormal behavior, loss of appetite, or unusual stool over the course of the study. Vaginal pH was measured prior to IVR insertion, during IVR use, and following IVR removal (Fig 1). The pH differences with the IVR in place and post removal were found to be insignificant from the baseline using a Friedman test with Dunn’s post-test for multiple comparisons (Day -6) and Wilcoxon matched-pairs signed rank test (Day 0). The median vaginal pH prior to IVR insertion and with the IVR in place was 8.50 (IQR, 8.20–8.85) and 8.50 (IQR, 8.50–8.70), respectively.

### Summary of drug concentration measurements

Drug and drug metabolite concentrations in key anatomic compartments are summarized in Table 4. Vaginal tissue drug samples were not collected with Configuration B pod-IVRs in place, but were collected on Day 31, one day after IVR removal, to analyze drug washout.

**Table 4:** Summary of drug and drug metabolite concentrations at all sampled anatomic sites (six animals).

Analyte, matrix, units^a^	*N*^*b*^	% > LLOQ^c^	Proximal^d^^,^^e^	Distal^d^^,^^e^
**Configuration A**				
TAF,^f^ vaginal fluid, ng mg^-1^	24	12.5%	1.9 (NA)	0.5 (NA)
TFV, vaginal fluid, ng mg^-1^	24	100%	13.3 (4.2–17.4)	4.4 (1.9–7.1)
ACV, vaginal fluid, ng mg^-1^	24	100%	25.8 (10.3–46.2)	11.7 (3.6–21.0)
TFV, vaginal tissue, ng mg^-1^	6	100%	NA^g^	2.4 (1.2–7.8)
TFV-DP, vaginal tissue, fmol mg^-1^	6	83.3%	NA^g^	20.2 (12.6–97.2)
ACV, vaginal tissue, ng mg^-1^	18	100%	NA^g^	0.2 (0.1–0.3)
TFV, plasma, ng mL^-1^	12	91.7%	1.2 (0.7–2.0)	NA^g^
ACV, plasma, ng mL^-1^	12	91.7%	3.98 (3.19–4.25)	NA^g^
ENG, plasma, ng mL^-1^	12	100%	2.00 (1.46–2.29)	NA^g^
EE, plasma, ng mL^-1^	12	100%	0.10 (0.06–0.27)	NA^g^
**Configuration B**^**h**^				
TAF,^f^ vaginal fluid, ng mg^-1^	24	29.2%	0.2 (0.1–7.5)	0.75 (0.6–0.8)
TFV, vaginal fluid, ng mg^-1^	24	100%	5.7 (3. 8–9. 7)	3.9 (2.5–5.6)
ACV, vaginal fluid, ng mg^-1^	24	91.7%	28.7 (20.4–39.2)	20.0 (12.5–26.1)
TFV, plasma, ng mL^-1^	12	100%	0.6 (0.5–1.0)	NA^g^
ACV, plasma, ng mL^-1^	12	91.7%	3.31 (2.28–4.63)	NA^g^
ENG, plasma, ng mL^-1^	12	41.7%	0.42 (0.40–0.47)	NA^g^
EE, plasma, ng mL^-1^	12	100%	0.06 (0.05–0.08)	NA^g^

^a^Includes all values for timepoints with IVR in place

^b^Number of samples analyzed

^c^Proportion of samples that contained quantifiable drug levels

^d^Median (interquartile range, 25th - 75th)

^e^Relative to IVR

^f^analyzed as the free-base, not the hemifumarate salt (TAF_2_)

^g^NA, not applicable

^h^Unpaired *t* tests with Welch’s correction were used to compare CVF antiviral drug concentrations obtained with Configuration A *versus* Configuration B IVRs and found no statistically significant difference between these datasets in both proximal and distal sampling locations. The corresponding *P*-values are presented in the Results section.

### Cervicovaginal fluid drug levels

**Fig 5:**
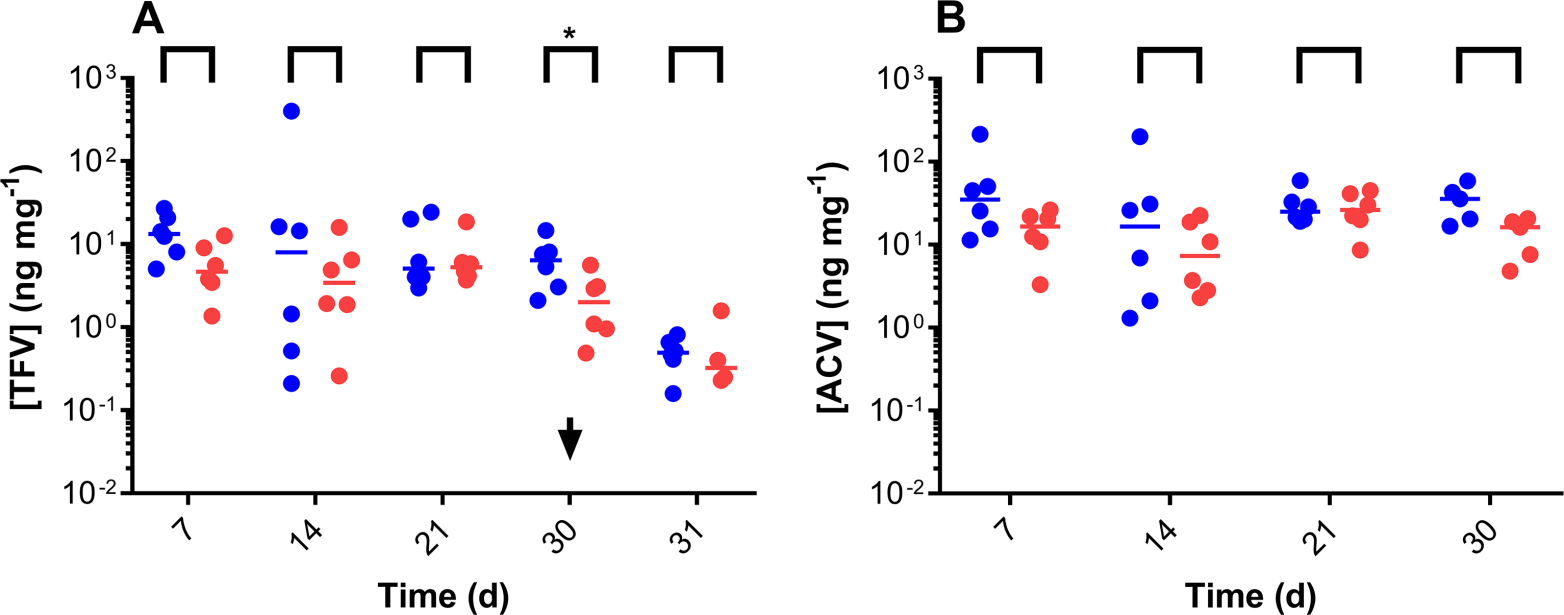
*In vivo* (*N* = 6) vaginal fluid drug levels from Configuration A (Day 7, Day 14) and Configuration B (Day 21, Day 30) TAF_2_-ACV-ENG-EE pod-IVRs. Blue, proximal to the IVR; red, distal to the IVR; each circle corresponds to an individual datum; horizontal lines correspond to medians. (A) TFV (TAF hydrolysis product) vaginal fluid concentrations. The arrow (Day 30) corresponds to the time of Configuration B IVR removal. Paired proximal and distal vaginal fluid TFV concentrations at each timepoint were compared using Wilcoxon matched-pairs signed rank test: Day 7, *P* = 0.1563; Day 14, *P* = 0.5625; Day 21, *P* > 0.9999; Day 30, *P* = 0.0313 (significantly different); Day 31 (washout), *P* = 0.8750. (B) ACV vaginal fluid concentrations. Paired proximal and distal vaginal fluid ACV concentrations at each timepoint were compared using Wilcoxon matched-pairs signed rank test: Day 7, *P* = 0.16; Day 14, *P* = 0.31; Day 21, *P* = 0.84; Day 30, *P* = 0.19.

Steady state concentrations of TFV (Fig 5A), the hydrolysis product of the prodrug TAF_2_, and ACV (Fig 5B) proximal to the pod-IVRs as a function of time were maintained in cervicovaginal fluid (CVF) over the 30 days of IVR use (one-way ANOVA; TFV, *P* = 0.4660; ACV, *P* = 0.8090). The concentrations of Met X and Y [41, 42] were below the LLOQ of the assay in these samples. Paired CVF TFV and ACV concentrations proximal and distal to the IVRs were not statistically different (Fig 5), with the exception of TFV on Day 30 (*P* = 0.0313). Unpaired *t* tests with Welch’s correction were used to compare CVF antiviral drug levels in Configuration A *versus* Configuration B: TFV, proximal, *P* = 0.3074; TFV, distal, *P* = 0.6669; ACV, proximal, *P* = 0.3751; ACV, distal, *P* = 0.0900. There was no significant difference between these paired datasets.

Quantification of ENG in vaginal fluid samples was complicated by the wide dynamic range afforded by the rapid- and slow-releasing IVRs. While ENG levels in Configuration A (rapid-releasing ENG) were all above the lower limit of quantitation, only 50% of samples–both proximal and distal to the IVRs–were above the upper limit of quantitation. The median quantifiable ENG concentrations in this configuration were 4.8 ng mg^-1^ (IQR, 0.3–10.9 ng mg^-1^). In Configuration B (slow-releasing ENG), 54% of samples–both proximal and distal to the IVRs–were below the lower limit of quantitation. The median quantifiable ENG concentrations in this configuration were 0.2 ng mg^-1^ (IQR, 0.1–0.3 ng mg^-1^).

The analysis of EE in vaginal fluid samples by ELISA appeared to suffer from an interference precluding accurate measurements. However, EE vaginal fluid levels were consistently higher with the IVRs in place and a concentration gradient was apparent, with higher levels proximal to the IVRs. Median paired proximal:distal EE concentration ratios with the IVRs in place were 3.6 (IQR, 2.1–5.5).

### Vaginal tissue drug concentrations

**Fig 6:**
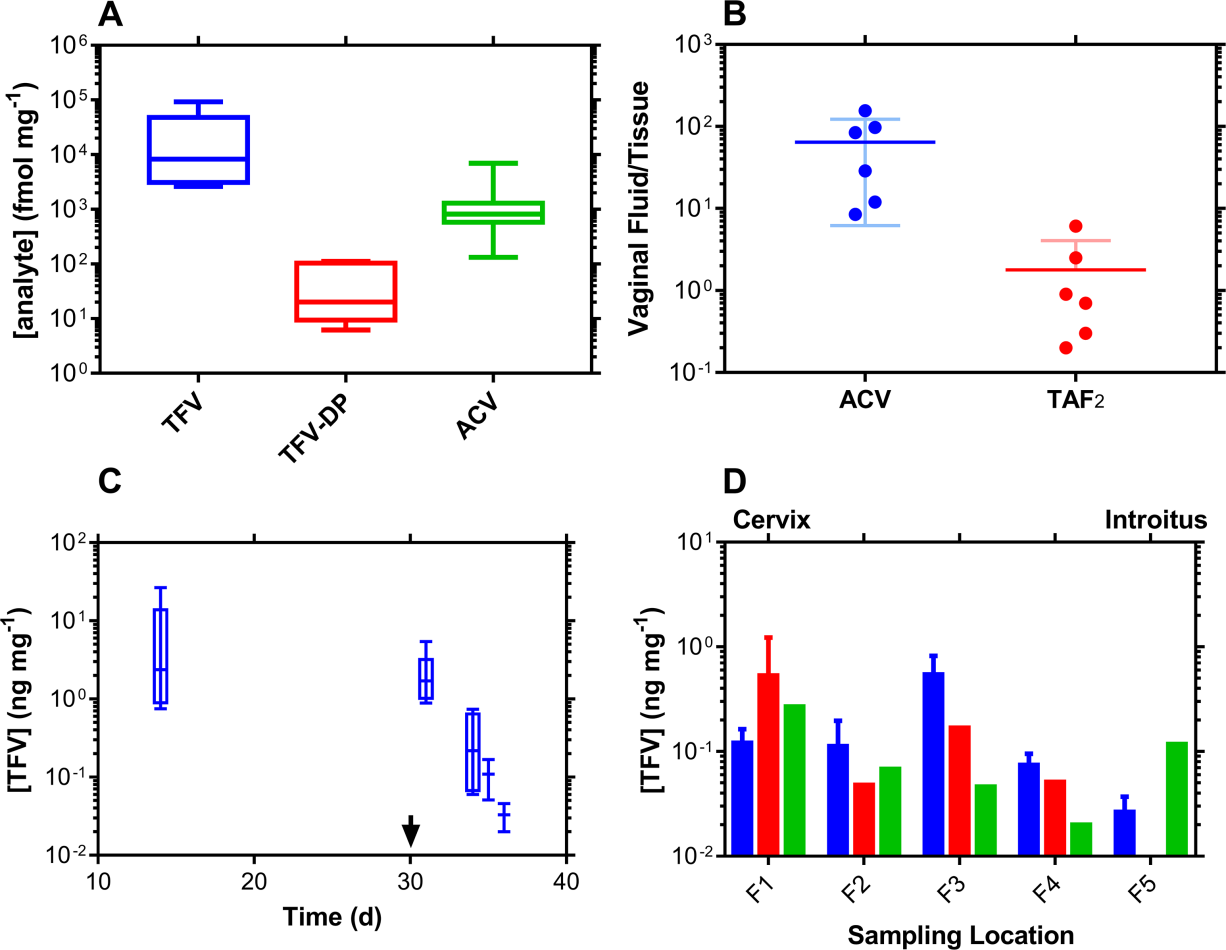
*In vivo* (*N* = 6) vaginal tissue homogenate drug levels from Configuration A and Configuration B TAF_2_-ACV-ENG-EE pod-IVRs. (A) Box plots of Day 14 (Configuration A) vaginal tissue molar concentrations at pod-IVR removal. The box extends from the 25th to 75th percentiles, with the horizontal line in the box representing the median; whiskers represent the lowest and highest datum. Vaginal tissue biopsies were collected distally to the pod-IVRs. Molar concentration units (fmol mg^-1^) were used on the y-axis to allow direct comparison between analytes. (B) Paired vaginal fluid:vaginal tissue concentration ratios of TFV (delivered as TAF_2_) and ACV at Day 14. The ratios provide a measure of the extent of tissue penetration for each analyte following vaginal delivery and, hence, vaginal bioavailability. (C) Box plots representing TFV washout from vaginal tissues following pod-IVR removal on Day 30 (arrow). The box extends from the 25th to 75th percentiles, with the horizontal line in the box representing the median; whiskers represent the lowest and highest datum. Vaginal tissue biopsies were collected on Day 14 and Day 31distally to the pod-IVRs. For Day 34–36 samples, collected at necropsy, only vaginal tissue TFV concentrations at locations F3 and F4 (see panel D) were used in the analysis to maintain consistency is sampling location (i.e., distal to the pod-IVRs). The median terminal half-life of TFV in vaginal tissues was found to be 18 h (IQR, 13–30 h). (D) Longitudinal distribution of TFV concentrations in whole vaginal tracts collected at necropsy (Day 34–36), i.e., following removal of the second set of IVRs. The vaginal tracts were divided into uniform segments (F1-F5) spanning from the cervix (F1, proximal to the IVR) to the introitus (F5) and the homogenized sections analyzed for TFV concentrations. Blue, Day 34 (*N* = 2); red, Day 35 (*N* = 2); and green, Day 36 (*N* = 2).

Molar antiviral drug concentrations in vaginal tissue biopsy homogenate are described in Fig 6A. These include the pharmacologically active metabolite of TFV (against HIV), tenofovir diphosphate (TFV-DP). The CVF (ng mg^-1^) to vaginal tissue (ng mg^-1^) drug concentration ratio (Fig 6B) provides a simple measure of xenobiotic partitioning between the two anatomic compartments: the lower the ratio, the more the antiviral agent distributes into the vaginal mucosa and the higher the vaginal bioavailability. The concentration ratio for TAF_2_ is approximately 40 times lower than for ACV.

The measurement of TFV levels post-IVR removal on Day 30 allows the drug washout kinetics to be analyzed (Fig 6C). A terminal half-life of elimination of 18 h (IQR, 13–30 h) was calculated from these data. The longitudinal (cervix to introitus) TFV distribution in vaginal tissue homogenate on three successive days during washout (i.e., after IVR removal) is shown in Fig 6D. No systematic pattern could be identified.

### Intracellular TFV-DP concentrations

**Fig 7:**
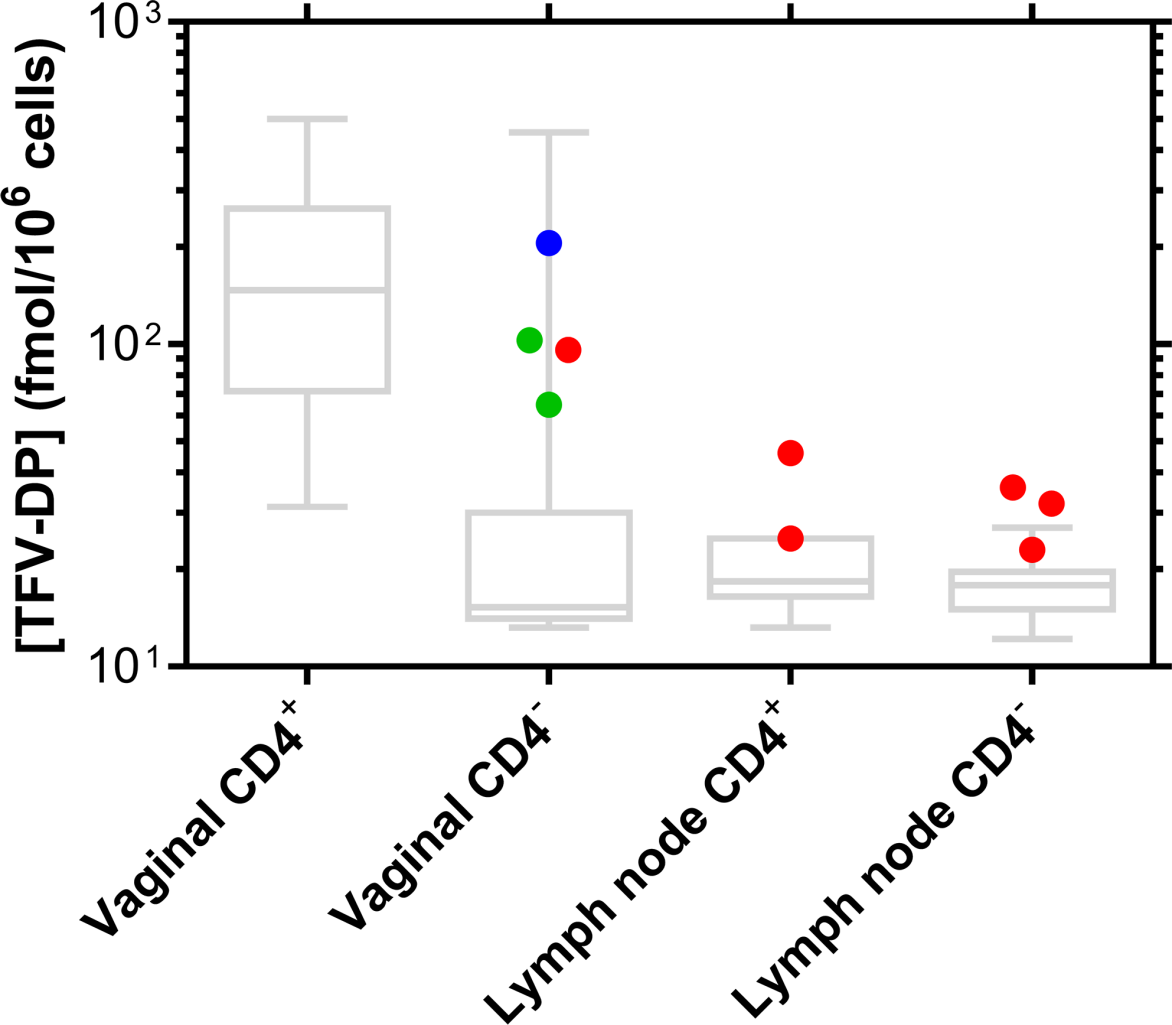
Intracellular TFV-DP concentrations in vaginal tissues and inguinal and iliac lymph nodes collected at necropsy. Blue, Day 34 (*N* = 2); red, Day 35 (*N* = 2); and green, Day 36 (*N* = 2). The intracellular TFV-DP levels are maintained 4–6 days after pod-IVR removal on Day 30. The box plots represent the analytical lower limits of quantitation (LLOQ) associated with the TFV-DP measurements, where the box extends from the 25th to 75th percentiles, with the horizontal line in the box representing the median; whiskers represent the lowest and highest datum. The assay has an LLOQ of 50 fmol per sample, which is divided by the number of cells collected in each sample to afford the individual LLOQs represented here.

Intracellular TFV-DP concentrations in CD4^+^ and CD4^-^ cells isolated from vaginal tissues and inguinal and iliac lymph node tissues at necropsy and during drug washout (Days 34–36, the IVRs were removed on Day 30) are presented in Fig 7. The small number of CD4^+^ cells isolated from vaginal tract sections precluded the quantitation of TFV-DP in these samples, as illustrated by the corresponding high LLOQ box plot in Fig 7.

### Systemic (plasma) drug concentrations

Systemic drug concentrations resulting from pod-IVR use are summarized in Table 4 for both formulations. Plasma TFV was quantified in 92–100%, depending on the pod-IVR formulation (Table 4). In previous studies on TFV or TDF IVR delivery in pigtailed macaques [39, 43, 50], plasma TFV concentrations usually are not quantifiable in most samples. However, the analytical methods for measuring plasma TFV in these prior studies had higher LLOQs (1 ng mL^-1^ or higher) than in our current study (0.31 ng mL^-1^, Table 2), possibly explaining the observation.

**Fig 8:**
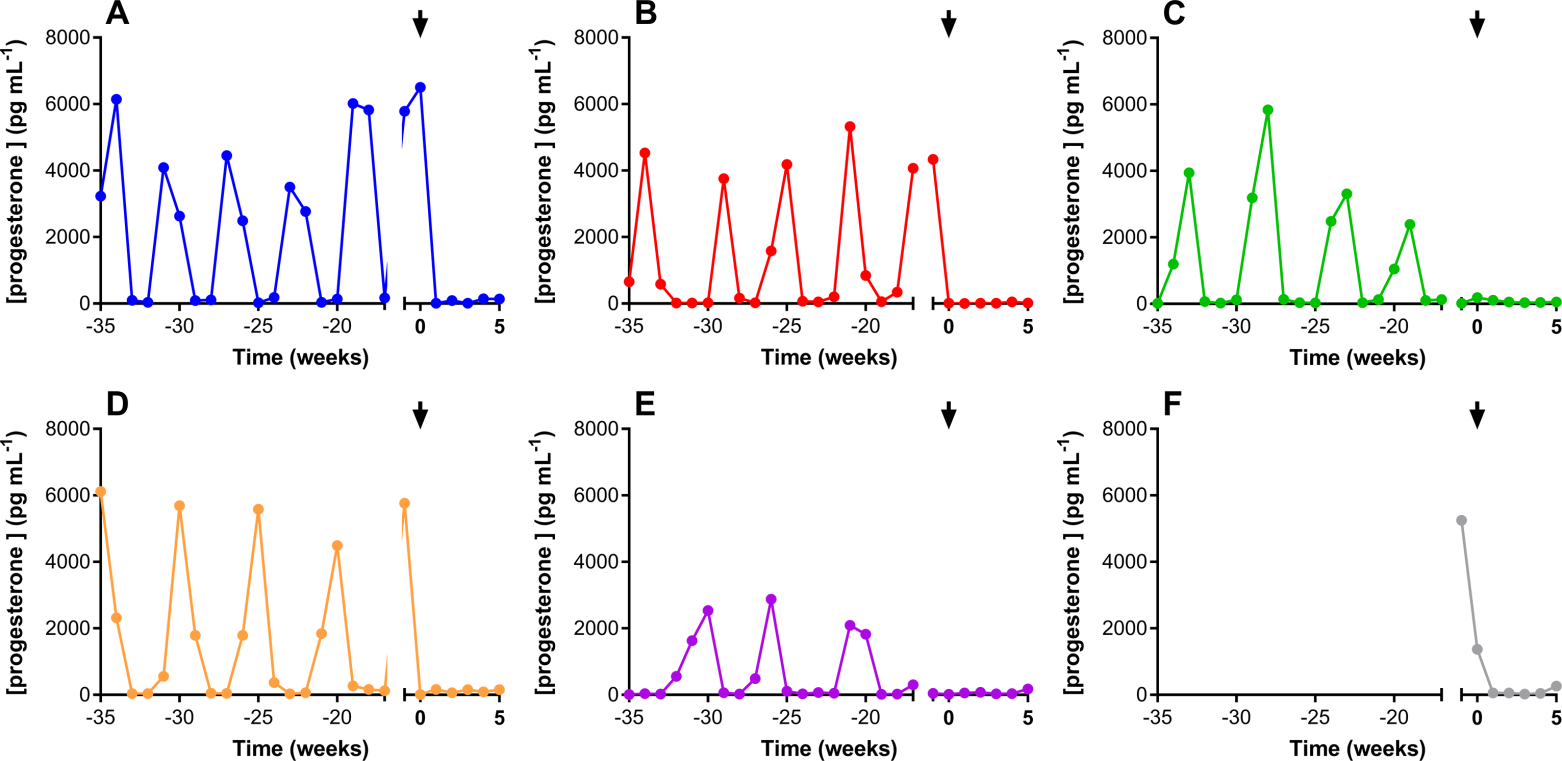
Plasma progesterone concentrations over 40-week period. Each panel represents one animal; the arrow indicates the placement of the Configuration A pod-IVR (week 0).

Plasma progesterone levels over a 40-week period are presented in Fig 8. Use of the Configuration A pod-IVRs started on Week 0, as indicted by the arrow in all panels. Endogenous progesterone production was effectively inhibited in all animals by the higher ENG-releasing IVR and maintained by the lower-releasing configuration.

## Discussion

The primary objective of the current study was to assess the PK in pigtailed macaques of a novel, multipurpose IVR for the prevention of HIV, HSV, and unintended pregnancy. We were able to utilize SHIV-infected macaques for a PK pilot study of short duration, followed by necropsy to obtain samples that are not feasible with a traditional PK design. Consequently, the IVRs were evaluated for ca. two weeks for each configuration. We acknowledge that a longer evaluation period, possibly spanning multiple, monthly pod-IVR changes, would have been beneficial. However, the time limitation for studies in infected animals scheduled for necropsy is restricted to limit pain and suffering and traditional, non-terminal PK studies are not amenable to the extensive tissue collection described here. Based on results from the current report, an expanded PK evaluation of the pod-IVR configuration described can be carried out in uninfected animals in future studies. The *in vivo* release rates, measured based on the residual drug content of the used devices, was less than 1 mg d^-1^ for all drugs (Table 3). A human-sized pod-IVR can accommodate up to 10 pods, ca. 45 mg each [36, 48]. Assuming two pods for the hormonal contraceptives and four pods for each of the antiviral agents, release rates well in excess of 1 mg d^-1^ would be feasible for one month while still remaining within the predicted linear release regime when 20% or more of the drug payload is present in the pods [48]. Given the low release of ENG (0.12 mg d^-1^) and EE (0.015 mg d^-1^) required for efficacy of the NuvaRing in humans, the highest required dosing would be for the two antiviral agents, and release rates of up to 5 mg d^-1^ would be feasible from a 28-day pod-IVR.

Rigorous PK testing in nonhuman primates constitutes a key preclinical evaluation in the development of vaginal drug delivery products. The pigtailed macaque model is particularly relevant because of its similarities with the human menstrual cycle, vaginal architecture and microbiome, and the ability to conduct efficacy studies with simian-human immunodeficiency virus (SHIV) [43, 47, 50–52]. The implications of our findings are discussed below in the context of developing a viable MPT candidate targeted at resource-poor regions.

### Biomedical product design considerations

Multipurpose IVRs will need to simultaneously deliver API combinations at independently controlled rates [37, 38]. Conventional IVR technologies–i.e., matrix and reservoir rings [26]–are based on API diffusion through the elastomer backbone that makes up the ring. This approach complicates the development of combination IVRs partially explaining why, to date, all such MPT IVRs have delivered two APIs [53, 54], with the exception of the MZCL IVR under development by the Population Council [55] and the pod-IVR described here [38]. We have developed an innovative pod-IVR platform that can simultaneously deliver multiple drugs in a modular fashion [36]. The polymer-coated drug cores, referred to as pods, are embedded in an unmedicated ring. This approach leads to a number of important benefits, discussed in detail elsewhere [26, 36]. In the context of combination IVRs as an MPT, the pod-IVR design readily enables the delivery of three or more drugs from a single device, as described in the literature [38, 39] and below.

### Adherence to IVR use

Two clinical trials were the first to evaluate the efficacy of an ARV IVR for HIV PrEP [33, 56]. Both found that a significant proportion of trial participants, particularly young women, did not adhere to study product use. These results raised concerns on the viability of IVRs for HIV PrEP in resource-limited regions. However, IVRs also are a new, female-controlled option in sub-Saharan Africa where contraceptive IVRs are not commonly used as in the developed world. Many believe [30, 31, 57–59] that adherence to IVR use for HIV PrEP will increase as familiarity with the devices increases and the trials transition into open-label phases. The same trend was observed with oral Truvada (TDF-FTC)/Viread (TDF) where adherence, and hence HIV PrEP efficacy, increased dramatically when moving from the initial, blinded, placebo-controlled trials to the open-label follow-on trials [20, 60–62].

The inclusion of a contraceptive component in MPT IVRs is believed by many to significantly motivate product adherence for HIV PrEP [30, 31, 57–59], thereby improving efficacy. The contraceptive component may be especially important for young women (18–21 years of age), the age group that was least adherent in the two phase 3 dapivirine IVR trials [33, 56]. However, the best approach is arguably to give women options that best suit their needs. For women that prefer no contraception, or a different contraceptive regimen (e.g., oral, injectable, or implant), the pod-IVR gives physicians additional versatility.

### The choice of antiviral agents and PK implications

The first MPT IVR for the prevention of HIV and HSV infection delivered a combination of TFV and ACV in the ovine model [37]. We have subsequently shown that the vaginal tissue bioavailability in sheep of the prodrug TDF was nearly 100 times higher than for the parent drug TFV [63]. To date, there have been no reports on the delivery of TAF (or TAF_2_) *via* IVR. Tenofovir alafenamide was approved by the FDA on November 5, 2015 for the treatment of HIV/AIDS and is being touted as the successor to TDF [64]. Because of its high potency against HIV [65], TAF is administered orally at a 30-fold lower dose (10 mg) than TDF (300 mg) and accumulates selectively in lymphatic tissue and immune cells, the targets for HIV replication, potentially also leading to a higher resistance barrier [66]. Tenofovir alafenamide has an excellent safety profile [67], with a much lower risk of kidney toxicity or bone density changes than TDF [68]. The high vaginal bioavailability of TAF in pigtailed macaques demonstrated here (Fig 4B) is an encouraging result.

In the present study, pod-IVRs delivered TAF_2_ in pigtailed macaques at a similar, albeit slightly lower, rate than TDF (Fig 4A) in a previous TDF-FTC pod-IVR study, also in pigtailed macaques [39]. The median TFV vaginal fluid levels from TAF_2_ delivery varied between 3.9 and 13.3 ng mg^-1^, depending on the IVR configuration and sampling location (Table 4). These values are considerably lower than corresponding median TFV concentrations (110–180 g mg^-1^, depending on the sampling site) following TDF delivery [39]. The median TFV (2.4 ng mg^-1^) exposure in vaginal tissue homogenate following TAF_2_ delivery also was considerably lower than corresponding median TFV concentrations (28 ng mg^-1^) resulting from TDF delivery [39]. Vaginal tissue homogenate TFV-DP concentrations were not measured in our previous study, so that comparison is not possible. The low CD4^+^ cell count in vaginal tissues prevented the washout kinetics of intracellular TFV-DP in these cell types to be measured. It should be noted that vaginal tissue TFV and TFV-DP concentrations have limited value as surrogates for the prediction of efficacy in HIV PrEP when comparing two different TFV prodrugs delivered topically. TAF delivers TFV more efficiently into PMBCs than TDF or TFV [69] and *in vitro* TAF was found to be >600-fold and 80-fold more potent than parent TFV in CD4^+^ T-cells and MDMs, respectively [70]. Concentrations of TFV and TFV-DP in vaginal tissue homogenate from TDF therefore may not be predictive of intracellular TFV-DP levels in HIV target cells resulting from topical TAF_2_ delivery, and *vice versa*.

The results of our studies do not definitively identify TAF_2_ or TDF as a superior candidate for vaginal HIV PrEP. Given the different drug distribution of the two TFV prodrugs, it is challenging to make meaningful efficacy predictions based on vaginal fluid drug levels. However, the above TDF-FTC pod-IVR was fully protective of vaginal SHIV infection in pigtailed macaques [52], but also included the delivery of FTC in addition to TDF. Smith *et al*. showed that a reservoir IVR delivering only TDF afforded full protection from infection in pigtailed macaques using the rigorous, repeat low dose SHIV challenge model [50]. Vaginal tissue TFV levels obtained with these IVRs were highly variable ranging between 0.1–100 ng mg^-1^, with means around 10 ng mg^-1^ well within the range observed here (median, 2.4 ng mg^-1^; IQR, 1.2–7.8 ng mg^-1^, Table 4). These results suggest that the TAF_2_ release rates from the IVR may be sufficient for SHIV protection in macaques, although they could easily be increased in a future study [48]. The vaginal TAF_2_ or TDF concentrations *via* IVR delivery required for protection from HIV infection in humans are unknown.

Acyclovir is a potent, FDA-approved antiviral agent for the treatment of HSV infections [71, 72]. While it has been suggested that TDF could be used vaginally for the simultaneous prevention of HIV and HSV [73, 74], ACV represents a clearly superior candidate for topical HSV prevention as it is 100-fold more potent *in vitro* [75]. Acyclovir exhibited a low vaginal bioavailability relative to TAF (Fig 4B), a result that was not unexpected given its oral bioavailability in the 10–20% range [76]. The low oral bioavailability of ACV led to the development of the *L*-valyl ester prodrug, valacyclovir (VACV), which was found to exhibit a three- to five-fold higher oral bioavailability clinically than the parent compound [76]. Valacyclovir is a substrate for intestinal and renal peptide transporters (PepT1 and PepT2), largely explaining its enhanced oral bioavailability relative to ACV [77, 78]. Unfortunately, PepT1 and PepT2 were found to be underexpressed in human vaginal tissues [79], explaining why ACV is being used at this stage of product development [37, 80, 81].

The ACV concentration required to reduce virus-induced cytopathic effect (CPE) by 50% (EC_50_) during *in vitro* studies using clinical HSV-2 isolates was 0.04–0.2 ng μL^-1^ [75, 82]. These values refer to ACV levels in the culture medium, not the concentrations in HSV-infected cells where ACV is selectively monophosphorylated [83]. Unlike HIV, HSV can infect vaginal epithelial cells. Median ACV concentrations in CVF–the appropriate correlate for *in vitro* EC_50_ values–achieved here were 50–100 times higher (Table 4) than the highest of the above EC_50_ values. The dose of ACV needed to suppress vaginal HSV replication or prevent acquisition in humans is unknown. A regimen of oral ACV (200 mg) taken five times daily for 10 days led to peak ACV concentrations in vaginal fluids in the 0.18–0.81 ng mg^-1^ range, 0.5–1 h after the final oral dose [84, 85], 14 to 165 times lower than the vaginal fluid ACV concentrations obtained here (median: 11.7–29.7 ng mg^-1^, depending on the IVR configuration and sampling location, Table 4).

### Hormonal contraception in MPT IVRs

In IVR-based hormonal contraception, progestin-estrogen combinations have demonstrated superior clinical efficacy to devices delivering only progestin [86]. Several large multicenter trials evaluating a matrix IVR delivering the progestin levonorgestrel (LNG) found pregnancy rates at 12 months between 3.7% [87] and 5.1% [88]. In contrast, the NuvaRing®, which delivers a combination of ENG and EE, demonstrated clinical efficacy in excess of 99% in Europe and in the United States [89–91]. The ENG:EE daily *in vivo* release ratios of Configuration A and Configuration B pod-IVRs were targeted to bracket the corresponding value obtained with the NuvaRing. These targets were achieved and plasma progestin levels during the 30 days of IVR use were effectively suppressed in all animals.

A number of MPT IVRs under development are based on progestin-only contraception using LNG [53–55]. There is growing concern, largely based on observational evidence from injectable depot medroxyprogesterone acetate (DMPA) [92], that an LNG-only regimen as part of an MPT may lead to an increased risk of HIV acquisition in women [93, 94]. More research is needed to further investigate the potential links between hormone exposure in the vaginal mucosa and STI susceptibility, including HIV.

In conclusion, topical administration of four drugs (one antiretroviral agent, one antiherpetic agent, and a contraceptive estrogen-progestin combination) from pod-IVRs in pigtailed macaques demonstrated preliminary safety while exhibiting sustained and controlled drug release over 30 days of consecutive product use. Given that all drugs achieved either drug concentrations associated with antiviral effect in other studies (TAF_2_, AVC) or demonstrated biologic activity in our study (ENG:EE suppressing progestin concentration), our pod-IVR holds significant potential for the prevention of vaginal HIV and HSV acquisition, along with unintended pregnancy, and merits further investigation.

## Supporting information

S1 File Detailed method descriptions for all new bioanalysis assays. (DOCX)
