# Supplementary material for: Novel multipurpose pod-intravaginal ring for the prevention of HIV, HSV, and unintended pregnancy: Pharmacokinetic evaluation in a macaque model
Source: PLoS One. 2017 Oct 5;12(10):e0185946. doi: 10.1371/journal.pone.0185946 (PMC5628903; doi:10.1371/journal.pone.0185946)
Supplement: S1 File — (DOCX) [file pone.0185946.s001.docx]

**Supporting Information**

**Novel multipurpose pod-intravaginal ring for the prevention of HIV, HSV, and unintended pregnancy: Pharmacokinetic evaluation in a macaque model**

James M. Smith,^#1^ John A. Moss,^#2^ Priya Srinivasan,^1^ Irina Butkyavichene,^2^ Manjula Gunawardana,^2^ Rob Fanter,^2^ Christine S. Miller,^2^ Debbie Sanchez,^2^ Flora Yang,^2^ Shanon Ellis,^3^ Jining Zhang,^3^ Mark A. Marzinke,^4,5^ Craig W. Hendrix,^4^ Amita Kapoor,^6^ and Marc M. Baum^*2^

^1^Laboratory Branch, Division of HIV/AIDS Prevention, **National Center for HIV/AIDS, Viral Hepatitis, STD, and TB Prevention,** Centers for Disease Control and Prevention, 1600 Clifton Rd NE, Atlanta, Georgia, United States of America

^2^Department of Chemistry, Oak Crest Institute of Science, 128-132 W. Chestnut Ave., Monrovia, California, United States of America

^3^Libra Management Group, 160 Clairemont Ave. Suite 200 Decatur, Georgia, United States of America

^4^Department of Medicine, Johns Hopkins University, 600 North Wolfe Street, Osler 500, Baltimore, Maryland, United States of America

^5^Department of Pathology, Johns Hopkins University, 1800 Orleans Street, Sheikh Zayed Tower, B1020-G, Baltimore, Maryland, United States of America

^6^Wisconsin National Primate Research Center, University of Wisconsin-Madison, 1223 Capitol Court, Madison, Wisconsin, United States of America

**Materials and methods**

**Cervicovaginal fluid bioanalysis**

For analysis of TAF_2_ in CVF, samples were thawed on ice and 100 µL aliquots were dispensed into 96-well plates, along with a minimum of six standards and a minimum of three quality controls prepared in the appropriate matrix in accordance with FDA guidelines [1]. Samples were spiked with 10 µL of internal standard (IS) solution (1 µg mL^-1^ MVC). Sample purification was carried out in a 96-well format using a protein and phospholipid removal system (Phree, Phenomenex, Inc., Torrance, CA) according to the manufacturer’s instructions. The purified samples were dried *in vacuo* using a SpeedVac concentrator system (Savant SC210A Plus, Thermo Fisher Scientific, Inc.) and were reconstituted in 0.1% (vol/vol) formic acid in water (100 µL) prior to analysis.

The concentrations of TAF (free-base) and its metabolites Met X and Met Y [2,3] in the processed CVF were measured by LC-MS/MS using a 5 μL injection volume and an HPLC system consisting of a model G1367A well-plate autosampler and a model G1312A binary pump (1200 Series, Agilent Technologies, Santa Clara, CA) operating at 0.8 mL min^-1^ interfaced to an API 3000 triple quadrupole tandem mass spectrometer (AB Sciex, Framingham, MA) with a Turbo Ion Spray electrospray ionization source. An Agilent Zorbax Eclipse XDB-C18 Rapid Resolution column (2.1 × 50 mm; 3.5 µm) controlled at 40°C was the stationary phase. The following gradient program was used (A, 0.1% vol/vol formic acid in water; B, 0.1% vol/vol formic acid in acetonitrile): 0.25 min 100% A; 1.25 min ramp from 100:0 A:B to 70:30 A:B; 1.0 min ramp from 70:30 A:B to 50:50 A:B; 0.5 min hold at 50:50 A:B; 2 min ramp from 50:50 A:B to 95:5 A:B; 0.5 min ramp from 95:5 A:B to 100:0 A:B resulting in a total run time of 5.5 min, with a TAF retention time of 2.40 min. The measured transition ions, *m/z*, under ESI+ ionization mode were: TAF, parent 477.1 amu, product, 270.5 amu; MVC (IS), parent 514.7 amu, product, 280.6 amu; TAF degradation product X (Met X), parent 401.1 amu, product 176.3 amu; TAF degradation product Y (Met Y), parent 359.1 amu, product 176.3 amu; TFV, parent 288.1 amu, product 176.2 amu.

For analysis of ACV in CVF, the following method was used. Samples were thawed on ice and 200 µL aliquots were dispensed into 96-well plates, along with a minimum of six standards and a minimum of three quality controls prepared in the appropriate matrix in accordance with FDA guidelines [1]. Samples were spiked with 10 µL of internal standard (IS) solution (1 µg mL^-1^ ACV-*d_4_*). Sample purification was carried out in a 96-well format using a solid phase extraction system (STRATA C18-E, Phenomenex, Inc., Torrance, CA) according to the manufacturer’s instructions. The purified samples were dried *in vacuo* using a SpeedVac concentrator system (Savant SC210A Plus, Thermo Fisher Scientific, Inc.) and were reconstituted in 0.1% (vol/vol) formic acid in water (100 µL) prior to analysis.

The concentration of ACV was measured by LC-MS/MS using a 5 μL injection volume and an HPLC system consisting of a model G1367A well-plate autosampler and a model G1312A binary pump (1200 Series, Agilent Technologies, Santa Clara, CA) operating at 0.8 mL min^-1^ interfaced to an API 3000 triple quadrupole tandem mass spectrometer (AB Sciex, Framingham, MA) with a Turbo Ion Spray electrospray ionization source. An Agilent Zorbax Eclipse XDB-C18 Rapid Resolution column (2.1 × 50 mm; 3.5 µm) controlled at 40°C was the stationary phase. The following gradient program was used (A, 0.1% vol/vol formic acid in water; B, 0.1% vol/vol formic acid in acetonitrile): 0.25 min 100% A; 1.25 min ramp from 100:0 A:B to 70:30 A:B; 1.0 min ramp from 70:30 A:B to 50:50 A:B; 0.5 min hold at 50:50 A:B; 2 min ramp from 50:50 A:B to 95:5 A:B; 0.5 min ramp from 95:5 A:B to 100:0 A:B resulting in a total run time of 5.5 min, with an ACV retention time of 0.3 min. The measured transition ions, *m/z*, under ESI+ ionization mode were: ACV, parent 226.2 amu, product, 152.2 amu; ACV-*d_4_* (IS), parent 230.2 amu, product, 152.2 amu.

For analysis of ENG in CVF, the following method was used. Samples were thawed on ice and 200 µL aliquots were dispensed into 96-well plates, along with a minimum of six standards and a minimum of three quality controls prepared in the appropriate matrix in accordance with FDA guidelines [1]. Samples were spiked with 10 µL of internal standard (IS) solution (0.5 µg mL^-1^ levonorgestrel, LNG). Sample purification was carried out in a 96-well format using a solid phase extraction system (STRATA C18-E, Phenomenex, Inc., Torrance, CA) according to the manufacturer’s instructions. The purified samples were dried *in vacuo* using a SpeedVac concentrator system (Savant SC210A Plus, Thermo Fisher Scientific, Inc.) and were reconstituted in 10% (vol/vol) methanol in water (100 µL) prior to analysis.

The concentration of ENG was measured by LC-MS/MS using a 5 μL injection volume and an HPLC system consisting of a model G1367A well-plate autosampler and a model G1312A binary pump (1200 Series, Agilent Technologies, Santa Clara, CA) operating at 0.45 mL min^-1^ interfaced to an API 3000 triple quadrupole tandem mass spectrometer (AB Sciex, Framingham, MA) with an atmospheric pressure photoionization source. An Agilent Poroshell SB-C18 column (2.1 × 150 mm; 2.7 µm) controlled at 40°C was the stationary phase. The following isocratic program was used (A, water; B, acetonitrile): 2 min 10% A, with an ENG retention time 1.2 min. The measured transition ions, *m/z*, under APPI+ ionization mode were: ENG, parent 325.1 amu, product, 257.5 amu; LNG (IS), parent 313.1 amu, product, 109.3 amu.

Cervicovaginal fluid EE levels were analyzed by ELISA using a commercial kit (Ecologiena® EE ELISA Kit, Tokiwa Chemical Industries Co., Ltd., Tokyo, Japan) according to the manufacturer’s instructions. Calibration curves in the 5-3,000 pg mL^-1^ range, with seven standards spanning the range, were employed.

**Vaginal tissue bioanalysis**

For analysis of ACV in vaginal tissue homogenate, the following method was used. Tissue specimens (vaginal biopsies or vaginal tract sections) were incubated with an enzyme cocktail (0.5 mL) consisting of collagenase (0.5 mg mL^-1^), DNase (2 U, Roche Diagnostics GmbH, Mannheim, Germany), elastase (12 U, Worthington Biochemical Corporation, Lakewood, NJ), hyaluronidase (60 U, Worthington Biochemical Corporation), and an internal control analyte. The digestions were carried out in Roswell Park Memorial Institute (RPMI) medium supplemented with 7.5% (vol/vol) fetal bovine serum (Thermo Fisher Scientific, Waltham, MA) in 1.5 mL microcentrifuge tubes at 37°C with vortex agitation every 15 min. Samples were macerated with a polypropylene micro pestle following incubation for 2.0 hours and re-incubated for an additional 30 min. The resulting digests were mixed by vortex agitation and centrifuged at 12,000×*g* for 10 min at 4°C. Aliquots (200 µL) of the resulting supernatant were purified immediately as described below.

Aliquots were dispensed into 96-well plates, along with a minimum of six standards and a minimum of three quality controls prepared in the appropriate matrix in accordance with FDA guidelines [1]. Samples were spiked with 10 µL of internal standard (IS) solution (1 µg mL^-1^ ACV-*d_4_*). Sample purification was carried out in a 96-well format using a solid phase extraction system (STRATA C18-E, Phenomenex, Inc., Torrance, CA) according to the manufacturer’s instructions. The purified samples were dried *in vacuo* using a SpeedVac concentrator system (Savant SC210A Plus, Thermo Fisher Scientific, Inc.) and were reconstituted in 0.1% (vol/vol) formic acid in water (100 µL) prior to analysis.

The concentration of ACV was measured by LC-MS/MS using a 5 μL injection volume and an HPLC system consisting of a model G1367A well-plate autosampler and a model G1312A binary pump (1200 Series, Agilent Technologies, Santa Clara, CA) operating at 0.85 mL min^-1^ interfaced to an API 3000 triple quadrupole tandem mass spectrometer (AB Sciex, Framingham, MA) with a Turbo Ion Spray electrospray ionization source. An Agilent Zorbax Eclipse XDB-C18 Rapid Resolution column (2.1 × 50 mm; 3.5 µm) controlled at 40°C was the stationary phase. The following isocratic program was used (A, 0.1% vol/vol formic acid in water; B, 0.1% vol/vol formic acid in acetonitrile): 1.5 min 75% A, with an ACV retention time 0.3 min. The measured transition ions, *m/z*, under ESI+ ionization mode were: ACV, parent 226.2 amu, product, 152.2 amu; ACV-*d_4_* (IS), parent 230.2 amu, product, 152.2 amu.

**Plasma bioanalysis**

For analysis of ACV in plasma, the following method was used. Samples were thawed on ice and 200 µL aliquots were dispensed into 96-well plates, along with a minimum of six standards and a minimum of three quality controls prepared in the appropriate matrix in accordance with FDA guidelines [1]. Samples were spiked with 10 µL of internal standard (IS) solution (0.1 µg mL^-1^ ACV-*d_4_*). Sample purification was carried out in a 96-well format using a solid phase extraction system (STRATA C18-E, Phenomenex, Inc., Torrance, CA) according to the manufacturer’s instructions. The purified samples were dried *in vacuo* using a SpeedVac concentrator system (Savant SC210A Plus, Thermo Fisher Scientific, Inc.) and were reconstituted in 0.1% (vol/vol) formic acid in water (100 µL) prior to analysis.

The concentration of ACV was measured by LC-MS/MS using a 5 μL injection volume and an HPLC system consisting of a model G1367A well-plate autosampler and a model G1312A binary pump (1200 Series, Agilent Technologies, Santa Clara, CA) operating at 0.8 mL min^-1^ interfaced to an API 3000 triple quadrupole tandem mass spectrometer (AB Sciex, Framingham, MA) with a Turbo Ion Spray electrospray ionization source. An Agilent Zorbax Eclipse XDB-C18 Rapid Resolution column (2.1 × 50 mm; 3.5 µm) controlled at 40°C was the stationary phase. The following gradient program was used (A, 0.1% vol/vol formic acid in water; B, 0.1% vol/vol formic acid in acetonitrile): 0.25 min 100% A; 1.25 min ramp from 100:0 A:B to 70:30 A:B; 1.0 min ramp from 70:30 A:B to 50:50 A:B; 0.5 min hold at 50:50 A:B; 2 min ramp from 50:50 A:B to 95:5 A:B; 0.5 min ramp from 95:5 A:B to 100:0 A:B resulting in a total run time of 5.5 min, with an ACV retention time of 0.3 min. The measured transition ions, *m/z*, under ESI+ ionization mode were: ACV, parent 226.2 amu, product, 152.2 amu; ACV-*d_4_* (IS), parent 230.2 amu, product, 152.2 amu.

For analysis of ENG in plasma, the following method was used. Samples were thawed on ice and 200 µL aliquots were dispensed into 96-well plates, along with a minimum of six standards and a minimum of three quality controls prepared in the appropriate matrix in accordance with FDA guidelines [1]. Samples were spiked with 10 µL of internal standard (IS) solution (0.1 µg mL^-1^ LNG). Sample purification was carried out in a 96-well format using a solid phase extraction system (STRATA C18-E, Phenomenex, Inc., Torrance, CA) according to the manufacturer’s instructions. The purified samples were dried *in vacuo* using a SpeedVac concentrator system (Savant SC210A Plus, Thermo Fisher Scientific, Inc.) and were reconstituted in 10% (vol/vol) methanol in water (100 µL) prior to analysis.

The concentration of ENG was measured by LC-MS/MS using a 5 μL injection volume and an HPLC system consisting of a model G1367A well-plate autosampler and a model G1312A binary pump (1200 Series, Agilent Technologies, Santa Clara, CA) operating at 0.45 mL min^-1^ interfaced to an API 3000 triple quadrupole tandem mass spectrometer (AB Sciex, Framingham, MA) with an atmospheric pressure photoionization (APPI) source. An Agilent Poroshell SB-C18 column (2.1 × 150 mm; 2.7 µm) controlled at 40°C was the stationary phase. The following isocratic program was used (A, water; B, acetonitrile): 2 min 10% A, with an ENG retention time 1.2 min. The measured transition ions, *m/z*, under APPI+ ionization mode were: ENG, parent 325.1 amu, product, 257.5 amu; LNG (IS), parent 313.1 amu, product, 109.3 amu.

For analysis of EE in plasma, the following method was used. Plasma samples (400-500 μL) were aliquoted into 15 mL glass extraction tubes. HPLC-grade water (500 μL, Fisher Scientific, Hampton, NH) was added to each tube followed by 50 μL of internal standard (EE-*d_4_*, 100 ng mL^-1^, CDN Isotopes, Pointe-Claire, QC). Internal standard was not added to double blanks. Extraction was performed by addition of 5 mL of 50:50 (vol/vol) ethyl acetate:hexane to all tubes which were then mixed by vortex agitation and centrifuged. The top (organic) layer was extracted into a clean glass test tube and dried in a water bath. EE was then derivatized by addition of 25 μL NaCO_3_ and 25 μL dansyl chloride (1 mg mL^-1^ in acetone) and heated for 3 minutes at 40°C. The sample was dried in a water bath and reconstituted in 100 μL 50% (vol/vol) acetonitrile and water for LC-MS/MS injection. A standard stock solution was prepared at 100 ng mL^-1^ in methanol and standards were diluted to create a 7-point calibration curve that ranged between 9.4-600 pg mL^-1^. Standards were reconstituted in blank rhesus serum and blanks and double blanks were created using blank rhesus serum.

Samples were analyzed on a QTRAP 5500 quadrupole linear ion trap mass spectrometer (SCIEX, Framingham, MA) equipped with a turbo spray ion source. The system included two Shimadzu LC20ADXR pumps and a Shimadzu SIL20ACXR autosampler. A sample of 10 μL was injected onto a Phenomenex Kinetex C18 column (2.1 × 100 mm; 2.6 µm) for separation using a mobile phase: water with 1% (vol/vol) formic acid (Solution A) and acetonitrile with 1% formic acid (Solution B), at a flow rate of 200 μL min^-1^. Sixty percent Solution B was held for 1 min followed by an increase to 90% Solution B for the next 1 min, then an increase to 98% B over 6 mins. This was held for 2 min before the system was returned to initial conditions of 60% Solution B and held for the final 3.1 min of each run. Mass spectrometer results were generated in positive-ion mode with the following optimized parameters: source temperature, 650°C; curtain gas, 35 psi; gas 1, 35 psi; gas 2, 40 psi; collisionally activated dissociation gas, medium. Quantitative results were recorded as multiple reaction monitoring (MRM) area counts after determination for the response factor for EE and EE-*d_4_* and the transitions were 530→171 and 534→171, respectively. The linearity of the calibration curve was *R^2^* > 0.9990 and the curve fit was linear with 1/x weighting. None of the compounds of interest were detected in blank or double blank samples. Intraassay coefficient of variation was determined by a low and high quality control pool of spiked EE into blank rhesus serum and ranged from 6-9%.

**References**

1. US FDA (2001) Guidance for Industry: Bioanalytical Method Validation. Rockville, MD: U.S. Department of Health and Human Services, Food and Drug Administration, Center for Drug Evaluation and Research (CDER), Center for Veterinary Medicine (CVM). 22 p.

2. Birkus G, Kutty N, He GX, Mulato A, Lee W, et al. (2008) Activation of 9-[(R)-2-[[(S)-[[(S)-1-(Isopropoxycarbonyl)ethyl]amino]phenoxyphosphinyl]-methoxy]propyl]adenine (GS-7340) and Other Tenofovir Phosphonoamidate Prodrugs by Human Proteases. Mol Pharmacol 74: 92-100.

3. Babusis D, Phan TK, Lee WA, Watkins WJ, Ray AS (2013) Mechanism for Effective Lymphoid Cell and Tissue Loading Following Oral Administration of Nucleotide Prodrug GS-7340. Mol Pharmaceut 10: 459-466.
